# Early Radiographic Warning Signs of Impending Lemierre's Syndrome

**DOI:** 10.7759/cureus.75976

**Published:** 2024-12-18

**Authors:** Richa S Nathan, Tyler J Ostrowski, Ananth Narayan, Neil Gildener-Leapman

**Affiliations:** 1 Otolaryngology - Head and Neck Surgery, Albany Medical College, Albany, USA; 2 Otolaryngology - Head and Neck Surgery, Albany Medical Center, Albany, USA; 3 Radiology, Albany Medical Center, Albany, USA

**Keywords:** head and neck infection, internal jugular vein thrombosis, lemierre's syndrome, radiographic findings, reactive lymphadenopathy, septic thrombophlebitis, social determinants of health

## Abstract

Lemierre’s syndrome is a rare and potentially life-threatening complication of head and neck infections, such as bacterial pharyngitis or tonsillitis. It is characterized by the extension of infection into the lateral pharyngeal spaces, leading to subsequent septic thrombophlebitis of the internal jugular vein(s). Although relatively uncommon since the advent of appropriate antibiotic therapy, the incidence of Lemierre’s syndrome has increased in the past 15 years, especially among young, healthy adults. This increase is likely attributed to the increasing prevalence of oropharyngeal infections in this population, making an initial diagnosis of Lemierre’s syndrome often elusive on presentation. Delayed recognition of this syndrome can result in treatment delays, increasing the morbidity and mortality in this condition. The diagnosis of Lemierre’s syndrome is typically confirmed through the identification of thrombophlebitis of the internal jugular vein on radiographic imaging and the isolation of anaerobic bacteria in blood cultures. Treatment involves prolonged antibiotic therapy and, often, anticoagulation.

This case report presents a rare complication of bacterial tonsillitis, with initial imaging that demonstrated left peritonsillar phlegmon and subtle micro-occlusions of the left internal jugular vein on early imaging. Within four days, the infection rapidly progressed to complete occlusion of the internal jugular vein with pulmonary septic emboli, culminating in Lemierre’s syndrome. This case highlights the importance of early detection and treatment of subtle radiographic findings of thromboses, along with consideration of social determinants of health, in the setting of head and neck infections to avoid rapid progression to Lemierre’s syndrome, a disease with an elusive initial presentation and potentially fatal outcomes.

## Introduction

Lemierre’s syndrome was first described in 1936 by Dr. Andre Lemierre in a case series of 20 patients with rare septicemias caused by anaerobic organisms [1]. He defined these cases of septicemia by a recent history of oropharyngeal infection, such as tonsillitis or pharyngitis, along with clinical or radiological evidence of internal jugular venous thrombosis, and anaerobic septicemia. *Fusobacterium necrophorum* was the bacterial species primarily responsible for these infections [1]. Lemierre initially named this syndrome “anaerobic post-anginal sepsis” due to the onset of sepsis occurring shortly after the patients had experienced a sore throat. It was not until the 1980s that this condition was routinely referred to as Lemierre’s syndrome [2]. With the advent of antibiotics in the 1940s and their widespread use for head and neck infections, the incidence of Lemierre’s syndrome decreased significantly. In fact, some clinicians even referred to it as a “forgotten disease” [3]. However, over the last 15 years, there has been an increase in the incidence of Lemierre’s syndrome especially among young, healthy adults, likely due to the higher incidence of oropharyngeal infections and antibiotic resistance in this population [4]. There is also a high rate of intravenous drug use, narcotic, and non-narcotic therapeutic drug use in this population, predisposing them to higher rates of oropharyngeal infections [4].

The pathogenesis of Lemierre’s syndrome occurs in three phases: a localized oropharyngeal infection, infectious extension to the parapharyngeal space of the neck with thrombophlebitis of the internal jugular vein, and septic emboli [2]. In addition to direct infection with *F. necrophorum* among other anaerobic pathogens, it is hypothesized that mucosal damage of the pharynx caused by other bacterial or viral infections leads to a fusobacterial superinfection, allowing for local infectious spread and subsequent widespread septicemia [2]. The release of septic emboli into the systemic circulation results in the widespread dissemination of bacteria into the lung, pleura, joints, bones, muscles, spleen, liver, kidney, and other circulatory endpoints [2]. This pathogenesis can occur for four to seven days. It can be very challenging to diagnose early as symptoms are usually non-specific and are often the same as those experienced in benign, self-limited pharyngeal infections [3]. A common clinical finding early in Lemierre’s syndrome is prolonged neck tenderness and swelling even after antibiotic treatment for pharyngitis [3].

The diagnosis of Lemierre’s syndrome begins with clinical signs and symptoms, but additional testing, such as imaging in the early stages of suspected Lemierre’s syndrome, can help confirm this diagnosis, which can be life-saving. Imaging is often conducted only once after septic emboli have developed in these patients [3]. In addition, even though blood cultures are indicated in this workup, they may be negative in up to 5% of cases due to the difficulties associated with culturing anaerobic organisms [3]. The treatment of Lemierre’s syndrome includes IV antibiotics, typically with broad-spectrum coverage such as beta-lactamase-resistant beta-lactam as an empiric therapy [3]. Other options include clindamycin or metronidazole for patients with beta-lactam allergies. Anticoagulation is sometimes indicated if thrombosis is extensive or causes significant symptoms [2]. This case presents a rare complication of bacterial tonsillitis, which initially revealed peritonsillar phlegmon and subtle micro-occlusions of the left internal jugular vein on early imaging. Within four days, the condition rapidly progressed to complete occlusion of the internal jugular vein with pulmonary septic emboli, leading to a diagnosis of Lemierre’s syndrome. This case highlights the importance of early detection and rigorous intervention in patients with head and neck infections who present with subtle radiographic findings of thrombosis. Timely management is essential to prevent the rapid progression of this often elusive and potentially fatal disease.

## Case presentation

This case study was conducted using chart review analysis of a 36-year-old male who presented to the emergency department with a chief complaint of pharyngitis, dysphagia, and odynophagia. He had been experiencing these symptoms for five days, with progressive worsening. The patient did not have any significant past medical history, but radiological imaging was ordered due to the severity and prolonged duration of his pain, which was out of proportion for a presentation of pharyngitis. In the emergency department (ED), the patient received three liters of IV lactated ringer’s solution, 20 mg of IV dexamethasone, along with 3.375 g IV of piperacillin/tazobactam and 600 mg IV of linezolid, which provided some symptomatic relief. The initial contrast-enhanced computed tomography (CT) images on the first presentation to the ED noted left peritonsillar microabscesses with phlegmon. Ipsilateral cervical reactive lymphadenopathy was also noted, with edema in the subcutaneous fat and submandibular gland. Surrounding inflammatory changes extended into the left parapharyngeal, masticator, and carotid spaces.

**Figure 1:**
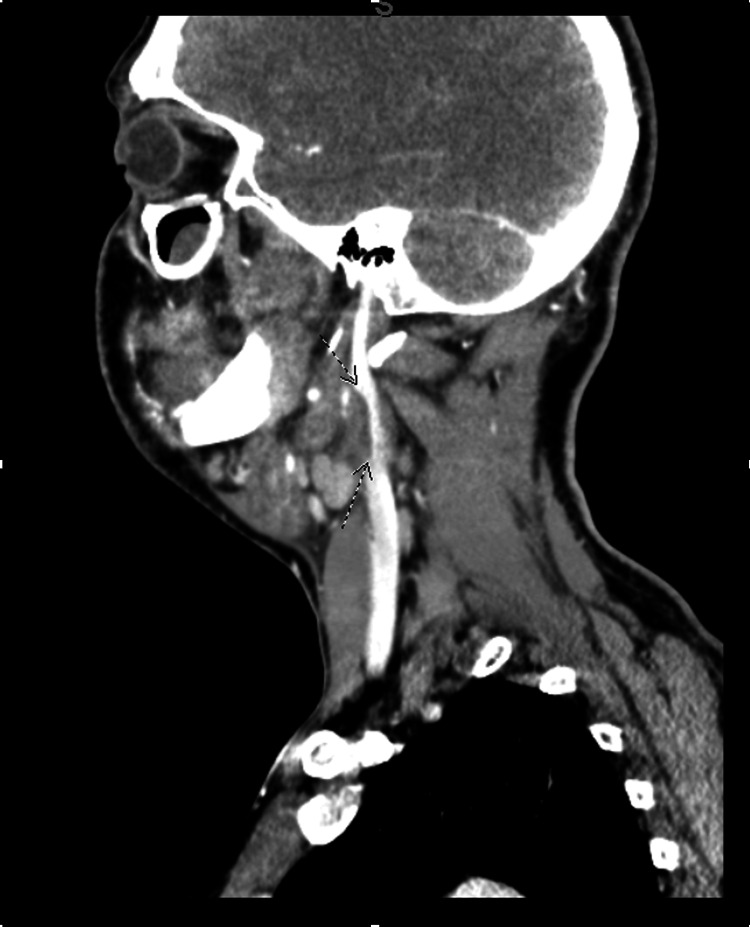
Initial sagittal contrast-enhanced CT scan demonstrating partial occlusion of the left internal jugular vein (IJV), as indicated by the arrows. The IJV with contrast filing defects suggests early signs of thrombophlebitis.

The initial sagittal CT scan showed a partial occlusion of the left internal jugular vein (IJV), as indicated by the arrows in Figure 1. The IJV was not filling normally with contrast, which suggested early signs of thrombophlebitis. The initial read of this scan did not reflect subtle micro-occlusion or IJV thromboses but was rather interpreted as peritonsillar phlegmon in the setting of pharyngitis. This image was consistent with the early stages of Lemierre’s syndrome, where jugular vein micro-thrombosis precedes local progression and eventual widespread dissemination of infection.

**Figure 2:**
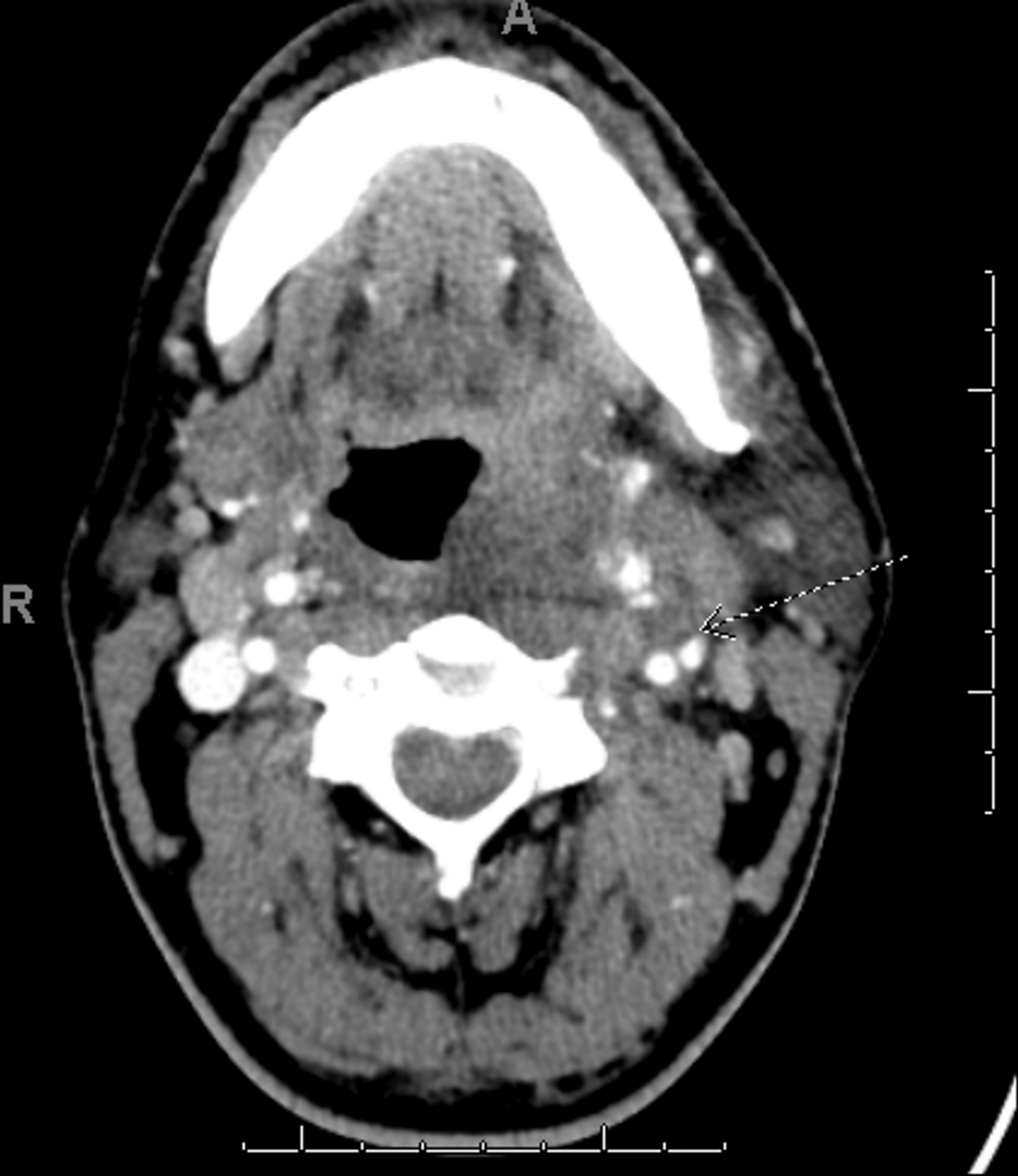
Initial contrast-enhanced axial CT scan showing internal jugular vein (IJV) filing defect indicating a partial occlusion of the left IJV as indicated by the arrow.

Figure 2 displays the initial contrast-enhanced CT scan from the axial view; the arrow indicates partial occlusion of the left IJV.

**Figure 3:**
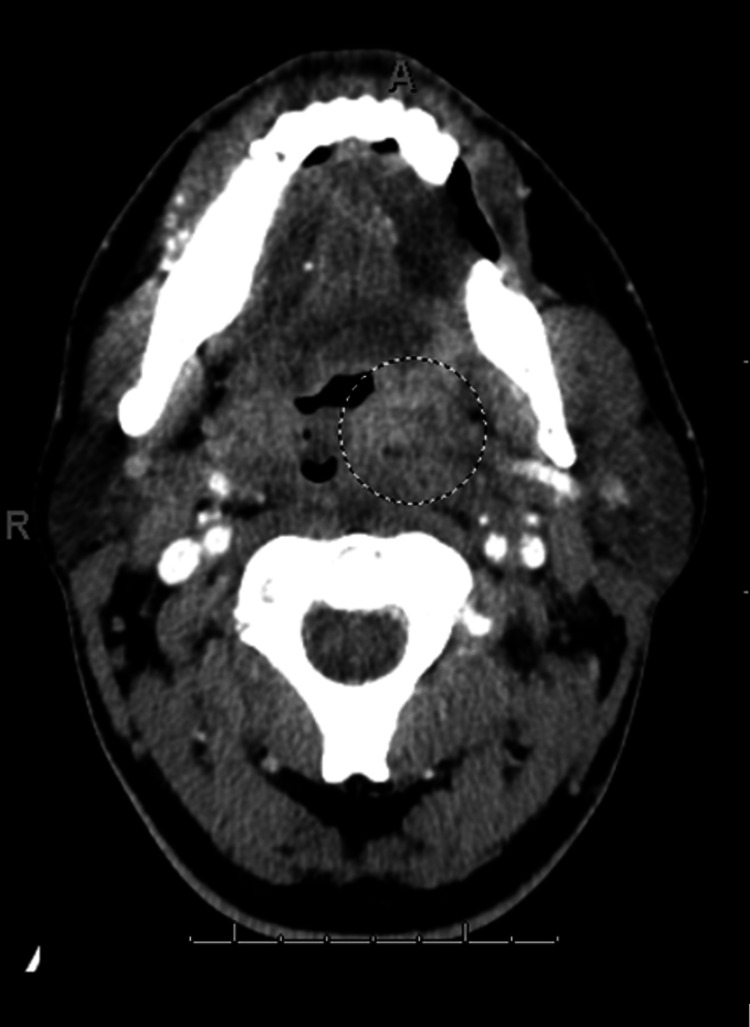
Initial axial contrast-enhanced CT image of the neck showing soft tissue swelling and edema in the oropharyngeal space, indicated by the circle. There is also enlargement of the left palatine tonsil.

Initial contrast-enhanced CT imaging also showed soft tissue swelling or edema in the oropharyngeal space. In addition, there was mild enlargement of the left palatine tonsil. This swelling represents inflammation, which was initially thought to be secondary to the normal phlegmon from pharyngitis. Figure 3 shows the axial contrast-enhanced CT scan, with the circle indicating the area of soft tissue edema.

**Figure 4:**
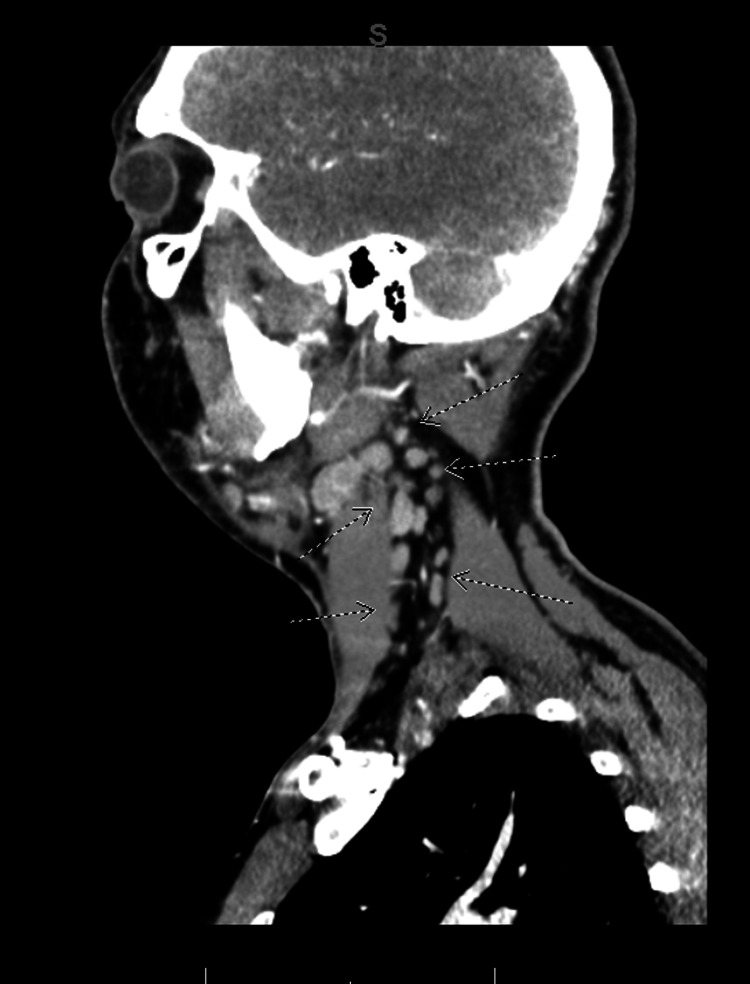
Initial sagittal contrast-enhanced CT with arrows indicating areas of soft tissue edema and reactive lymphadenopathy secondary to pharyngitis.

This same soft tissue edema is seen in the sagittal view in Figure 4. In addition to soft tissue edema, the arrows indicate areas of enlarged lymph nodes along the left side of the neck, indicative of reactive lymphadenopathy.

**Figure 5:**
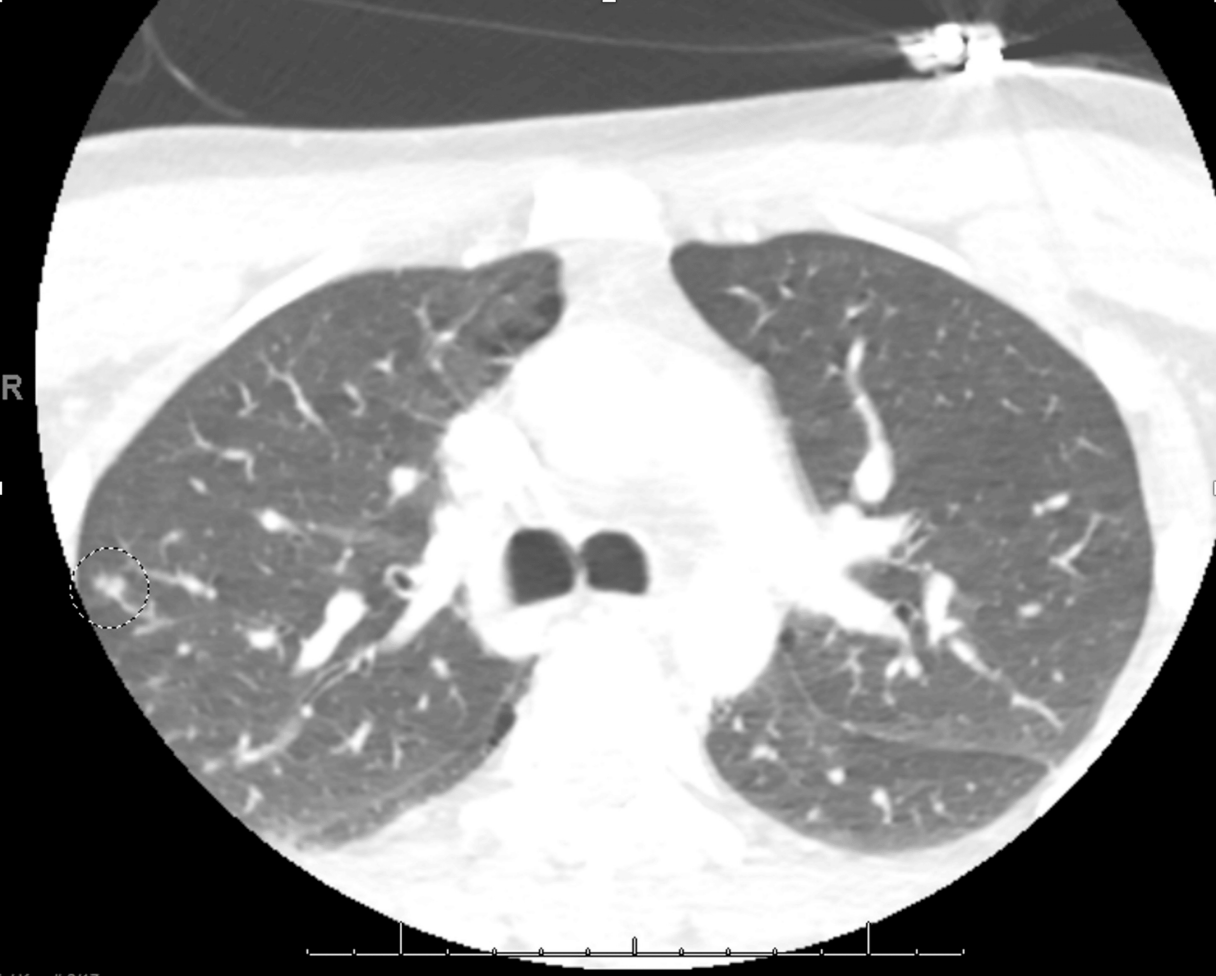
Initial contrast-enhanced CT axial image showing a small, well-defined nodule in the right lung (circled area) representing a septic embolus.

In addition, an initial axial contrast-enhanced CT scan of the lung parenchyma showed a small, well-defined nodule in the right lung (circled area), which represented an initial septic embolus, as seen in Figure 5. Septic emboli often appear as multiple nodular opacities scattered in the lung fields. They may cavitate as they evolve.

A diagnosis of tonsillitis with soft tissue cellulitis was made after analyzing the patient’s initial CT scan and symptoms. The patient was then discharged and prescribed 875 mg of amoxicillin/clavulanate every 12 hours for 7-10 days. Initial and repeat blood cultures came back negative and did not grow any microorganisms after five days in culture. This led the team to choose a broad-spectrum antibiotic as an initial treatment modality.

**Figure 6:**
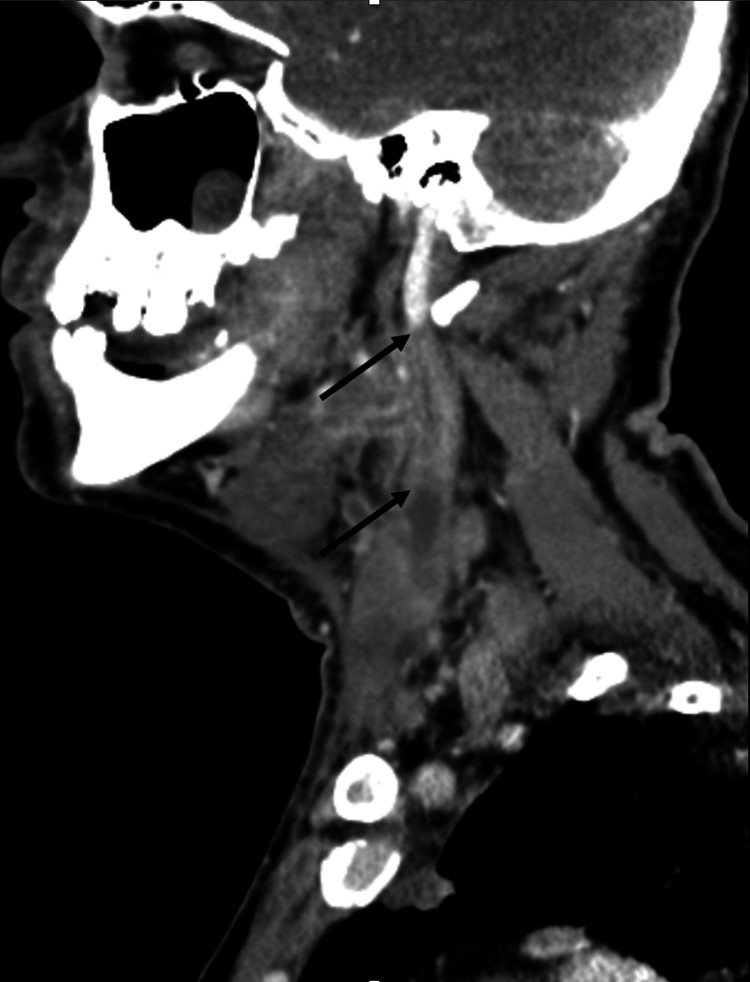
Repeat sagittal contrast-enhanced CT showing complete occlusion of left internal jugular vein indicated by the black arrows.

The patient re-presented to the ED one day after discharge with increasing left neck pain. This pain was associated with left-sided neck stiffness, trismus, fever, and chills. Upon this visit, the patient’s social history revealed that he was homeless, had a 20-pack-year smoking history, and had poor dental decay. Due to his social circumstances, he was unable to pick up the prescribed Augmentin the day before, which contributed to the progression of his disease course. In the ED, the patient was persistently hypotensive with systolic blood pressure in the 90s and met sepsis criteria. He was febrile, tachycardic with a heart rate of 130, and significantly dehydrated with labs reflective of acute kidney injury. The patient was admitted to the ICU for sepsis secondary to tonsillitis; his pain was consistently at a 10/10 in severity, and the patient was extremely nauseous. He reported additional symptoms of left shoulder pain along with neck pain, chest pain, and abdominal pain with cough and shortness of breath. Repeat CT neck was obtained (five days after primary imaging). Sagittal CT showed complete occlusion of the left IJV along with the facial, lingual, and anterior jugular veins, as seen in Figure 6 indicated by the black arrows.

**Figure 7:**
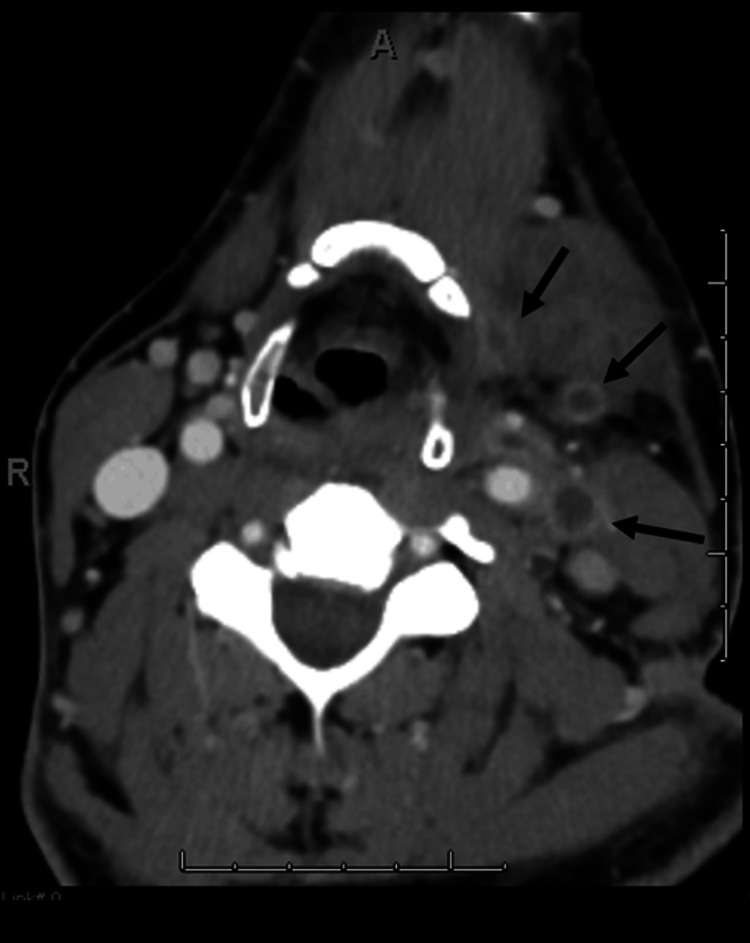
Repeat axial contrast-enhanced CT showing complete occlusion of the facial, lingual, and anterior jugular veins, indicated by the black arrow.

**Figure 8:**
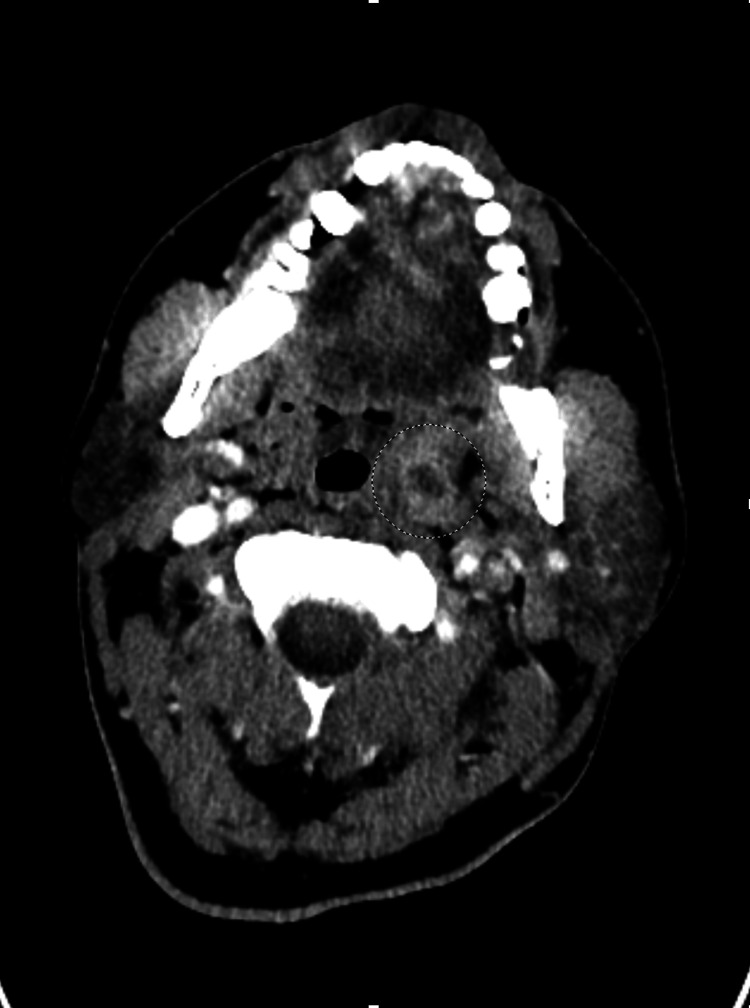
Repeat axial contrast-enhanced CT imaging showing the formation of a 1x1 cm peritonsillar abscess.

Figure 7 shows the axial contrast-enhanced CT slices, reflecting the complete occlusion of the left facial, lingual, and anterior jugular veins, indicated by the black arrow. Figure 8 shows an axial CT view of a 1x1 cm left peritonsillar abscess, indicated by the black circle.

**Figure 9:**
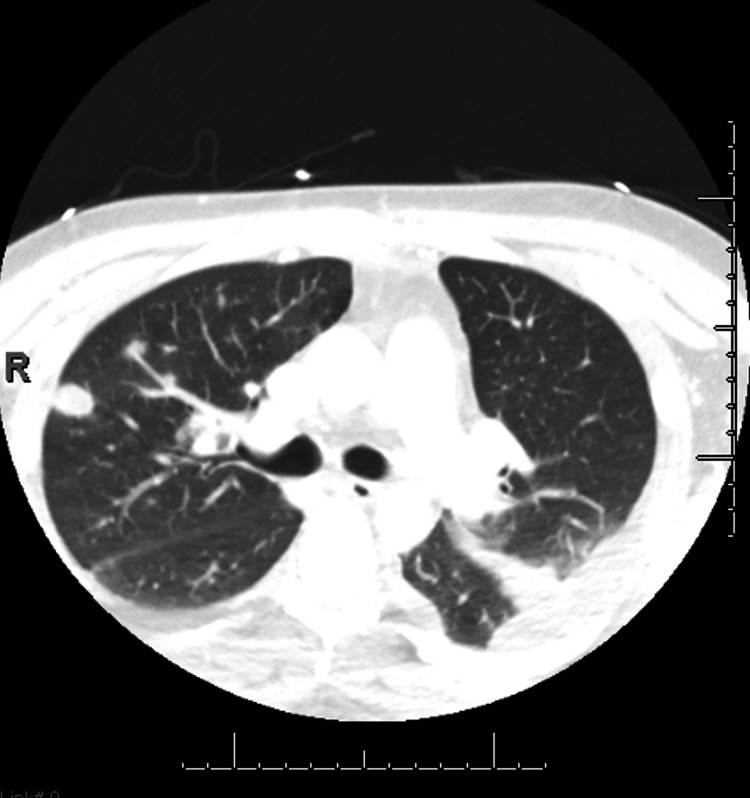
Repeat axial CT imaging showing extensive septic emboli

In addition, extensive pulmonary septic emboli were present bilaterally upon this second presentation. Figure 9 shows the axial chest CT slice of the lung parenchyma with septic emboli. There are multiple nodular opacities with several small, rounded nodules scattered throughout both lungs. Septic emboli often appear as nodules due to the emboli lodging in the pulmonary vasculature and causing localized inflammation and infection [5]. In addition, these nodules are located toward the periphery of the lungs, which is typical for septic emboli as they follow the pulmonary arterial tree and tend to lodge in the smaller, distal vessels [5]. The septic emboli originate from the infected thrombus in the internal jugular and collateral veins and spread to the lungs through the venous system. In this case, the septic emboli are the result of hematogenous dissemination of infected material.

Based on this repeat imaging, it was confirmed that the patient was experiencing sepsis secondary to a left IJV thrombus, reflective of Lemierre’s syndrome. His condition was further complicated by acute kidney injury, abnormal liver function levels, mild anemia, thrombocytopenia, and transaminitis in the setting of sepsis. A bilateral tonsillectomy was scheduled and performed on day 10 after the initial presentation. He was discharged from the hospital that same week with no complications and was directed to follow a soft, cool diet, continue 875 mg of amoxicillin/clavulanate every 12 hours for 7-10 days, and hold anticoagulation medication for one week postoperatively. He was scheduled for a two-week follow-up with the outpatient otolaryngology clinic at Albany Medical Center.

## Discussion

In hindsight and upon the patient’s discharge, it was noted that there were early radiographic warning signs for Lemierre’s syndrome in this patient. The initial CT neck showed subtle micro-occlusions of the left IJV that were not specifically noted initially. Had these findings been detected early on, and had the clinical team been more acutely aware of the significance of these findings indicating a potentially rapid progression to Lemierre’s syndrome, the patient would not have been discharged and may not have rapidly progressed to the potentially fatal complete occlusion of the left IJV.

Early detection and treatment of Lemierre’s syndrome can be difficult due to its initial non-specific symptoms, often resembling common oropharyngeal infections. Zhao et al. described a case of a 25-year-old male who initially presented with a sore throat, abdominal pain, and flu-like symptoms and was discharged with azithromycin and prednisone [6]. His condition worsened, prompting another ED visit, where he was found to have enlarged tonsils, cervical lymphadenopathy, and leukocytosis. Despite negative blood cultures and CT findings, his clinical course suggested Lemierre’s syndrome, which progressed to sepsis and septic emboli caused by *F. necrophorum*. This patient, similar to this case, highlights how the disease may present differently in each patient and underscores the importance of maintaining high suspicion, particularly when symptoms worsen despite treatment. Early imaging and intervention are crucial in managing this potentially fatal condition.

Riordan et al. performed a literature review involving five case series of Lemierre’s syndrome and analyzed the major features found in each case. IJV thrombosis was found to be present in only 26%-45% of all cases, while positive anaerobes grew from blood cultures in 97%-100% of those cases [7]. Despite the main diagnostic criteria for Lemierre’s syndrome being the detection of IJV occlusion, this is not reliable. Eilbert et al. described a patient with Lemierre’s syndrome who did not have positive blood cultures throughout their disease course but rather only dysphagia and non-specific symptoms of pharyngitis that rapidly progressed to complete occlusion of the IJV on CT [8]. This further highlights the importance of early imaging in suspected cases of Lemierre’s syndrome to avoid delaying treatment.

Dalen et al. mentioned that the key to early detection and treatment of impending Lemierre's syndrome is multifactorial. It includes recognizing the classic presentation of Lemierre's syndrome as that of young adult patients with a recent oropharyngeal infection presenting with sepsis, unilateral neck pain from internal jugular thrombophlebitis, and clinical or radiologic evidence of septic emboli [9]. Then, the isolation of *Fusobacterium *species should prompt a diagnosis of Lemierre's syndrome. The mainstay of treatment is the administration of intravenous antibiotics and empiric treatment that should cover anaerobic organisms, *Streptococcus *and *Staphylococcus *species, and include a β-lactamase inhibitor, such as piperacillin-tazobactam, as was started as an initial treatment in this case [9].

Since there is currently no standard diagnostic criterion for the diagnosis of Lemierre’s syndrome, providers must rely on their clinical judgment and proper interpretation of early imaging in detecting and treating Lemierre’s syndrome, especially considering the rarity of this condition and possible variants. This specific patient’s scenario also highlights the critical role social determinants of health play in disease progression. The patient’s inability to obtain his prescribed antibiotics due to his socioeconomic status significantly contributed to the rapid escalation of his condition. As medical providers, addressing barriers to medication access and timely follow-up, especially in underserved populations, is of crucial importance in preventing disease progression and improving patient outcomes.

## Conclusions

In conclusion, this case emphasizes the importance of detecting subtle findings of thromboses in initial radiological imaging when treating head and neck infections, such as tonsillitis. Clinical signs of neck stiffness, immobility, and aggressive pain should raise concern for a more challenging course than tonsillitis alone. It is also very important to consider every patient’s social determinants of health, which can predispose them to a more fulminant disease course when treatment is interrupted, leading to poor overall health outcomes. This case is unique in terms of the serendipitous capture of the progression of Lemierre’s syndrome, which has not been seen before. This patient’s elusive initial radiographic findings of micro-occlusions of the IJV and phlegmon rapidly progressed to sepsis with multi-organ involvement, characteristic of Lemierre’s syndrome.
